# Delphi methodology for symptomatology associated with visual dysfunctions

**DOI:** 10.1038/s41598-020-76403-9

**Published:** 2020-11-10

**Authors:** Mario Cantó-Cerdán, Pilar Cacho-Martínez, Ángel García-Muñoz

**Affiliations:** Departamento de Óptica, Farmacología y Anatomía, Universidad de Alicante, Apartado 99, 03080 Alicante, Spain

**Keywords:** Refractive errors, Epidemiology, Vision disorders

## Abstract

To analyse what eyecare clinicians think about which symptoms are associated with refractive, accommodative and binocular dysfunctions, and which of them should be used in a questionnaire of visual symptomatology. A Delphi method was developed, using a coordinating group and a group of experts, and the process was conducted in three rounds. In the first round we compiled a list of 34 symptoms from the scientific literature and additional 10 suggested by the experts. These symptoms were categorized by each expert to the associated visual anomalies and working distance. In the second round, the relationship between each symptom and visual dysfunctions was analysed using a numeric scale. In the third round, the appearance or absence of the 44 symptoms in a questionnaire was assessed. Symptoms most frequently assigned by the experts to visual anomalies were related to near vision. Symptoms of blurred vision, difficulty focusing from one distance to another and close one eye obtained the highest mean score for refractive, accommodative and binocular disorders respectively. The experts were in agreement for 15 symptoms and in disagreement for 5 symptoms that should appear in a questionnaire. Delphi method has been used to identify the symptoms related to visual dysfunctions according to eyecare professionals and has allowed to arrive at appropriate symptoms to be asked for in a visual symptomatology questionnaire.

## Introduction

The presence of any uncorrected visual dysfunction (refractive, accommodative or vergence anomaly) may lead to an increase in visual symptoms which could impact on patient’s visual comfort during the task. As symptoms may worsen with increased visual task, intervention will be necessary. The appropriate treatment of these visual problems may minimize or eliminate these symptoms.

These visual dysfunctions are commonly found in patients who attend optometry clinical practice^1–3^ although there is disparity according to the symptomatology they produce and which symptoms are considered for their diagnosis^4^. There are also differences in both the way of asking for symptoms and how the severity of the symptoms are categorised^5^. Sometimes clinicians ask for symptoms using questionnaires or by means of the clinical history. When using clinical history, there are also differences, as several practitioners analyse symptoms using patient´s descriptions of their case histories and in other situations symptoms are analysed on the basis of questions posed by the person who conducts the examination. Even when questionnaires are used, differences are showed as they use different scales to graduate the severity of symptoms. In any case, these differences do not influence the prescription of an appropriate treatment for any case^6^.

Recently, a systematic review^5^ has shown the wide disparity of symptoms related to different visual anomalies (refractive, accommodative and binocular ones) in the scientific literature and the differences between authors in methods for collecting this information. Most common symptoms may include *blurred vision, diplopia, headache* or *visual fatigue*, being 34 categories of symptoms, most of which are associated with near vision (Table 1). García-Muñoz et al.^5^ also found 11 different questionnaires for analysing visual symptoms^7–18^. Some questionnaires use dichotomous responses options and others by using multiple choice up to five responses for item. Nevertheless, only three questionnaires are psychometrically validated^8,9,14,15^. Two of them are only related to convergence insufficiency (Convergence Insufficiency Symptoms Survey (CISS) for children^14^ and adults^15^ and Convergence Insufficiency and Reading Study (CIRS) parent version^8^. The Conlon survey^9^ measures visual discomfort but it is not associated with any particular visual anomaly (refractive, accommodative or binocular).

**Table 1:** 34 symptoms categories shown in García-Muñoz et al.^6^ review.

Symptoms
Asthenopia
Avoid near tasks
Be distracted
Blurred vision
Change reading distance
Close one eye
Difficulty focusing from one distance to another
Difficulty performing schoolwork
Diplopia
Dry or gritty eyes
Excessive blinking
Excessive sensitivity to light
Extraordinary reading or writing posture
Eye turn noticed
Feel sleepy
Get faint colours around words
Head or book tilt
Headache
Inability to estimate distance accurately
Lack of concentration
Loss of place when reading
Ocular pain
Pulling eyes
Reading problems
Red eye
Rubbing of eyes
School performance problems
Sore eyes
Stomach ache or nausea
Tearing
Visual discomfort
Visual fatigue
Watery eyes
Words appear to move or jump at near vision

Due to the lack of availability of a generic symptom questionnaire to assess visual dysfunctions, this study aimed to use an approach to detect symptoms associated with visual anomalies which could be included in a questionnaire that would serve this purpose of aiding anomaly classification and measuring symptoms severity. Thus, clinicians could use this survey as an aid for diagnosing purposes and to monitor their treatment. For that, the first step would be to know which symptoms the clinicians would consider to be related to visual anomalies. And the Delphi method may be an efficient way to collect this information.

The Delphi technique is a formal consensus method used extensively in health care^19,20^ considered as an efficient way to collect information from a group of experts^19^. It is defined as a "method of structuring a group communication process that is effective in allowing a group of individuals, as a whole, to deal with a complex problem"^21–23^. It is a prospective structured communication technique, essentially to obtain qualitative information by taking into consideration the opinion of each member of the panel.

The Delphi method allow experts to communicate their opinions and knowledge about a complex problem in order to explore options and potentially reach consensus even if they are in geographically dispersed areas^24^. In fact, it has been shown that Delphi method is an useful tool to reach consensus in an area of uncertainty or lack of empirical evidence^25^. This method has several characteristics which make it different from other consensus methods^19^: response anonymity (members do not know each other, which allows them not to be inhibited in their responses); iteration (the process occur in rounds where interactions between group members is made via questionnaire rather face to face communication) and controlled individual feedback (showing the distribution of the group’s response). In addition to that, there is a statistical analysis using summary measures of the full group response. There is no standard method to calculate the number of experts for the Delphi technique. A sample of about fifteen has been suggested although larger panels may be used^26^.

Taking into account these considerations, for our research, the application of the Delphi method may be useful to assess the opinion of clinicians regarding which visual symptoms may be related to visual anomalies. It was chosen over other consensus techniques because its ability to allow all experts equal participation and influence. The scientific evidence^5^ shows that the available questionnaires have not been developed taking into account the experience of the optical and clinical set-up practitioners. And in any case have been developed using formal consensus methods as the Delphi method. So that using a Delphi method to know which symptoms are considered by the experts to assemble a robust questionnaire related to visual disorders, may be an important aid based in scientific evidence.

Therefore, the aim of this study is to identify which symptoms optometrists consider to be highly associated with presence of refractive, accommodative or binocular vision disorder, and of these symptoms, which ones should be included in a visual symptom questionnaire for diagnostic or treatment use.

## Methods

This research was approved by the ethical committee of the University of Alicante. All methods were carried out in accordance with relevant guidelines and regulations.

### Participants

For this study, we developed the Delphi method by establishing the coordinating and the expert group. The coordinating group comprised the authors of this manuscript. All of them were experienced optometrists and experts in public health. They were responsible for the overall coordinating of the study logistics. The coordinating role embraces functions such as modifying the study protocol where necessary, recruitment of experts, preparing the questionnaires of each round, analysing their responses as well as, interpreting the results from the study.

The experts’ group were responsible for giving their opinions in each round. We initially invited 17 Spanish optometrists to participate in the study. All of them were professionals of recognised prestige with more than 15 years of experience, and with expertise in the field of evaluation and treatment of different vision anomalies. Three of them rejected the invitation, so the final panel of experts consisted of 14 members. They were selected according to the main characteristics the scientific literature recommends^27^ to consider an expert for a Delphi technique, that is, according to their knowledge (several of them had a higher degree) and their experience (all of them had also clinical experience in several areas of vision). Of them, 5 optometrists developed their clinical practice in ophthalmological clinics and the other 9 in optical establishments so that this allowed to have different point of views from experts who examine different type of patients from their practices. All experts attended all age groups and different areas of visual health in their clinical practices. In this way, the group of experts included in the study covered different population groups. They were individually contacted via email in order to preserve anonymity. All experts gave their acceptance by informed consent to participate in this research.

### Design and procedure

We designed a specific database to collect the information obtained from the panel of experts. As experts were from different geographic areas, this method allowed to get different ideas from several optometrists from all over Spain. Due to anonymity, possible negative influences of the dominant members of the group or the inhibition of a participant were avoided^28,29^.

The coordinating group formulated the research question involved to the panel of experts and our application of the Delphi method involved three rounds. For each round there was an information exchange between the coordinating group and each expert individually. After each of them, the coordinating group collected the judgments and opinions of the experts and analysed them. Based on the responses collected and the interpretation of the coordinating group, a new questionnaire of the proposed problem was presented for the following round. As the coordinating group sent an abstract of the results obtained at the end of each round, before starting the next round, the experts knew the opinions obtained from all of them so that they could restate their answers depending on the results obtained in each round. With this procedure, a feedback was created through the analysis of the coordinating group, which allowed the flow of information among the experts and facilitated the establishment of a common language in each of the rounds. In addition, this approach allowed the experts the possibility of reflecting or reconsidering their opinion through their own approaches or from other experts as they could see the new information in the following round.

This procedure was done for each round until the coordinating group informed the experts of the panel’s conclusions. Each round lasted two weeks. If the participants did not respond at this time or did not complete the questionnaire, they were sent a reminder email, thus avoiding biases in the study. All data was performed using the SPSS 20.0 statistical package.

#### First round

In the first round, we compiled a list of 34 symptoms (Table 1) from a systematic review^5^ about symptoms and visual anomalies. In this round, we developed a closed response questionnaire and experts were requested to classify the 34 symptoms according to the anomalies (refractive, accommodative, or binocular) which they are most likely to be associated with. The experts had also to classify these symptoms according to whether they thought they could be associated with distant or near vision. This association was considered when the answers were designated by more or equal than 50% of experts. Furthermore, in this first round the experts suggested to add 10 symptoms to the initial 34. These symptoms were considered when at least one expert recommended one of them. The 10 additional symptoms included: *clumsy/stumble with objects; difficulty completing assignments on time; fixation difficulty; forgetful; inability to maintain demanding visual effort activities continuously; irritability with visual effort activities; mist/spiderweb; neck and or back pain; pain in the area of the eyebrows* and *squint to see better*. Once the results were received, they were synthesised, analysed and sent to the experts together with the necessary documents to start the second round.

#### Second round

In this round the experts answered a closed response questionnaire in which each of them had to quantify the relationship between the 44 categories of symptoms and each of the dysfunctions. They had to rate the relationship by a scale of 0–10, with 0 being the least related and 10 the most related. Once all the answers were received, they were synthesised and analysed (by means of the mean and standard deviation), and results of this round were again sent to the experts together with the third circulation.

#### Third round

A closed answer questionnaire was also used. The experts had to indicate whether each of the 44 symptoms could appear in a visual symptom questionnaire. For this purpose, we used a Likert scale of 5 responses, where the experts had to answer their level of agreement to include each symptom in the questionnaire (*strongly disagree*/*disagree*/*neither agree or disagree*/*agree*/*strongly agree*). They had only to point out one of the specific responses for each symptom. When all responses were received, they were synthesised and analysed (by means of median and the interquartile range exposed through a box and whisker plot), and the third round results were sent back to the experts. Consensus for symptoms being in a questionnaire was assumed (by de coordination group) when the median was in the level of *strongly agree* in addition to have all scores between *neither agree or disagree* and *strongly agree*. Using this criterion, we can assure that no one expert disagrees with the inclusion of a symptom in the questionnaire. Consensus for symptoms not being in questionnaire was assumed when the median was in the level of *strongly disagree*, *disagree* and *neither agree or disagree*.

**Figure 1:**
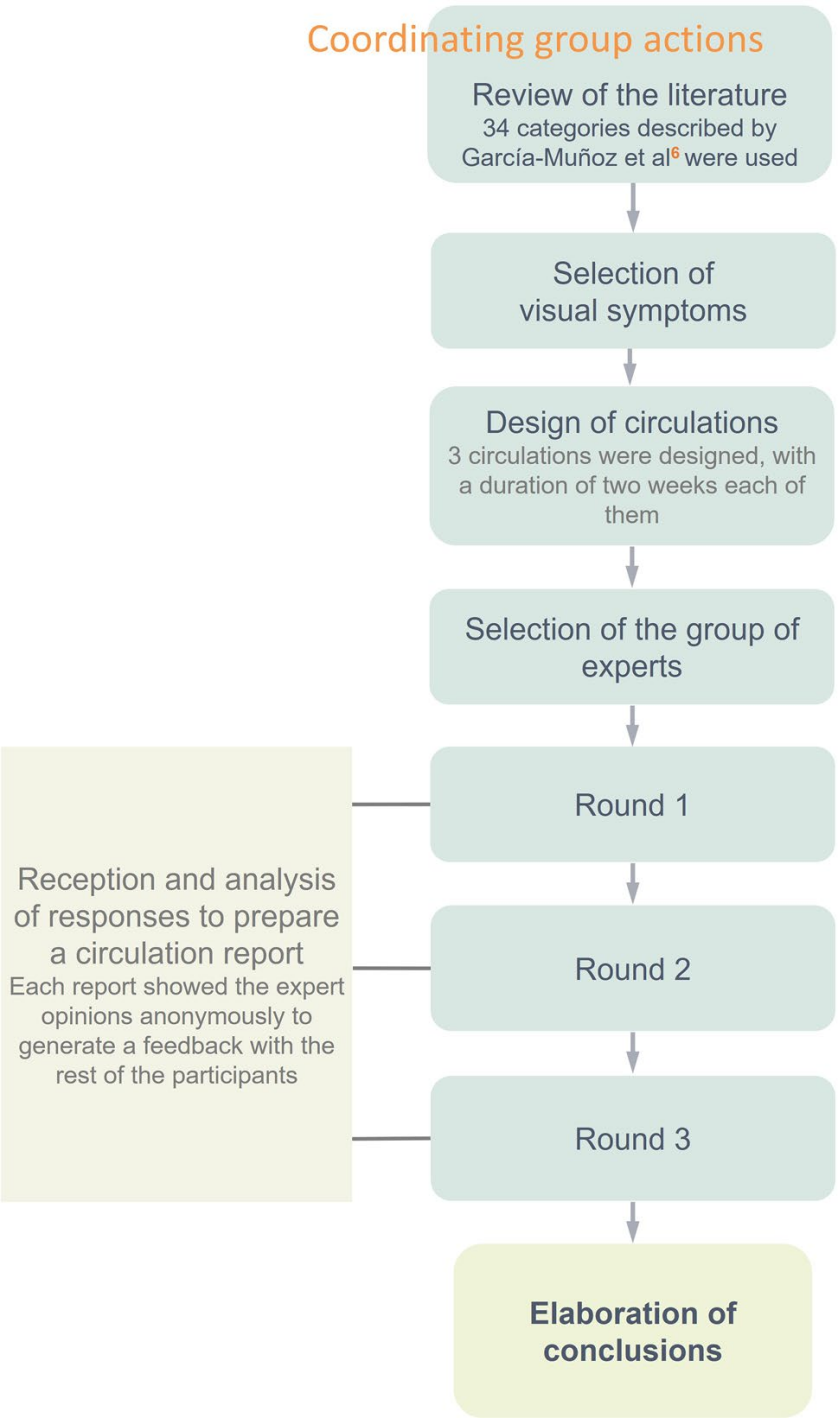
Representation of Delphi methodology used in the study.

Afterwards, all data of the three rounds were analysed and a final report was revealed to the experts with the conclusions of the panel. Figure 1 shows the Delphi method developed by the coordinator group.

## Results

**Figure 2:**
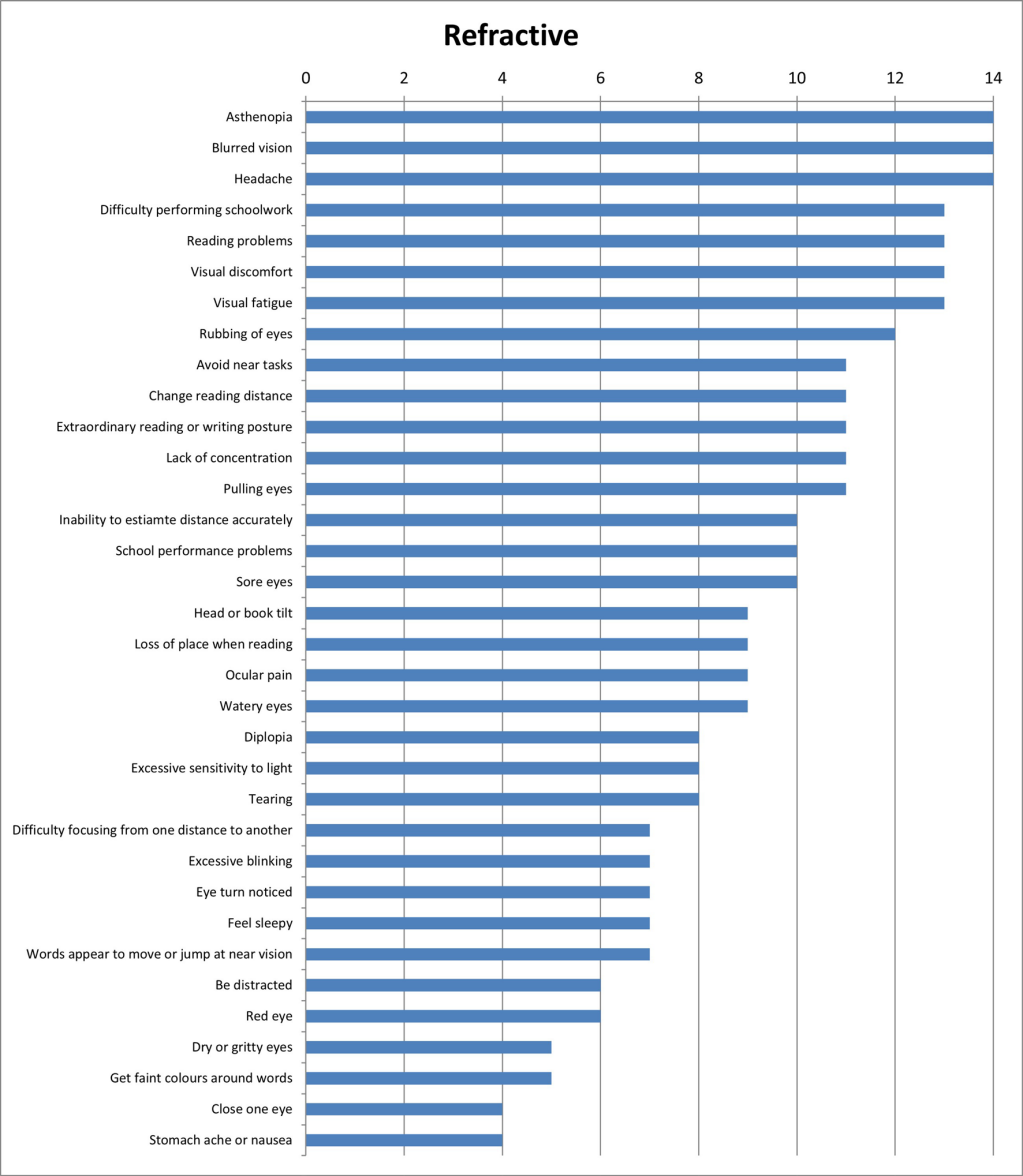
Results of round 1 for refractive dysfunctions. Number of experts who associate each of 34 initial symptoms with refractive dysfunctions.

**Figure 3:**
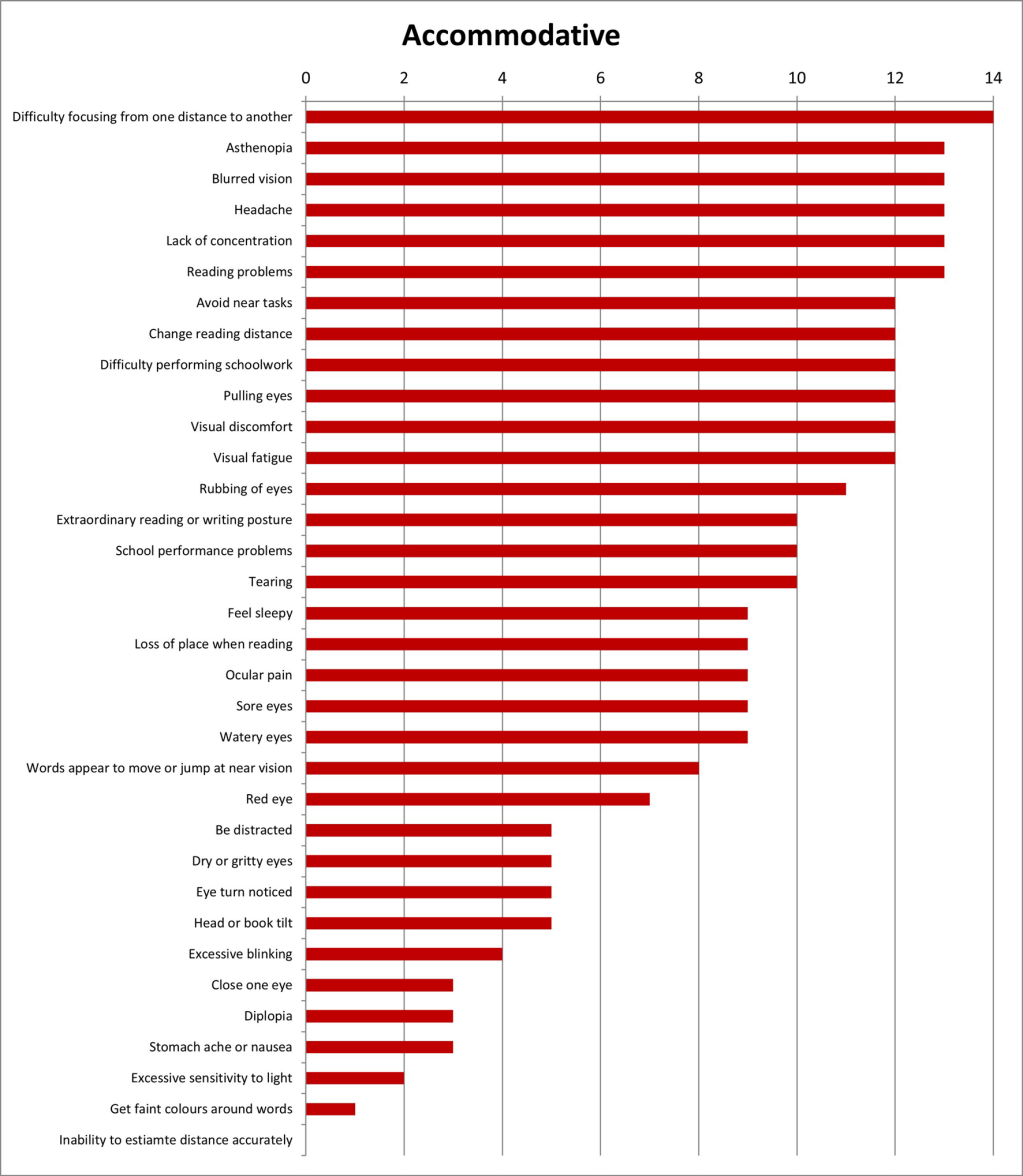
Results of round 1 for accommodative dysfunctions. Number of experts who associate each of 34 initial symptoms with accommodative dysfunctions.

**Figure 4:**
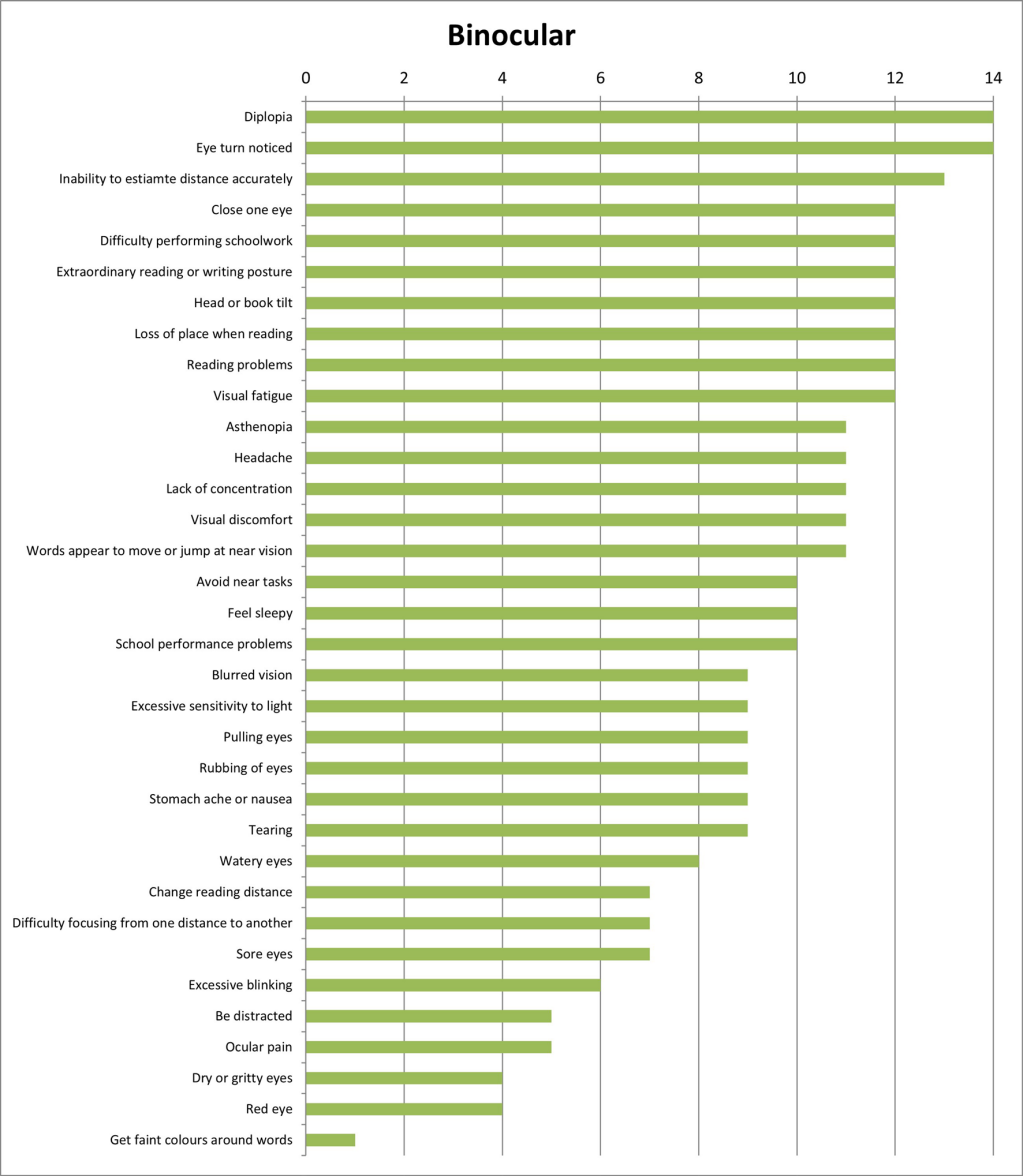
Results of round 1 for binocular dysfunctions. Number of experts who associate each of 34 initial symptoms with binocular dysfunctions.

**Figure 5:**
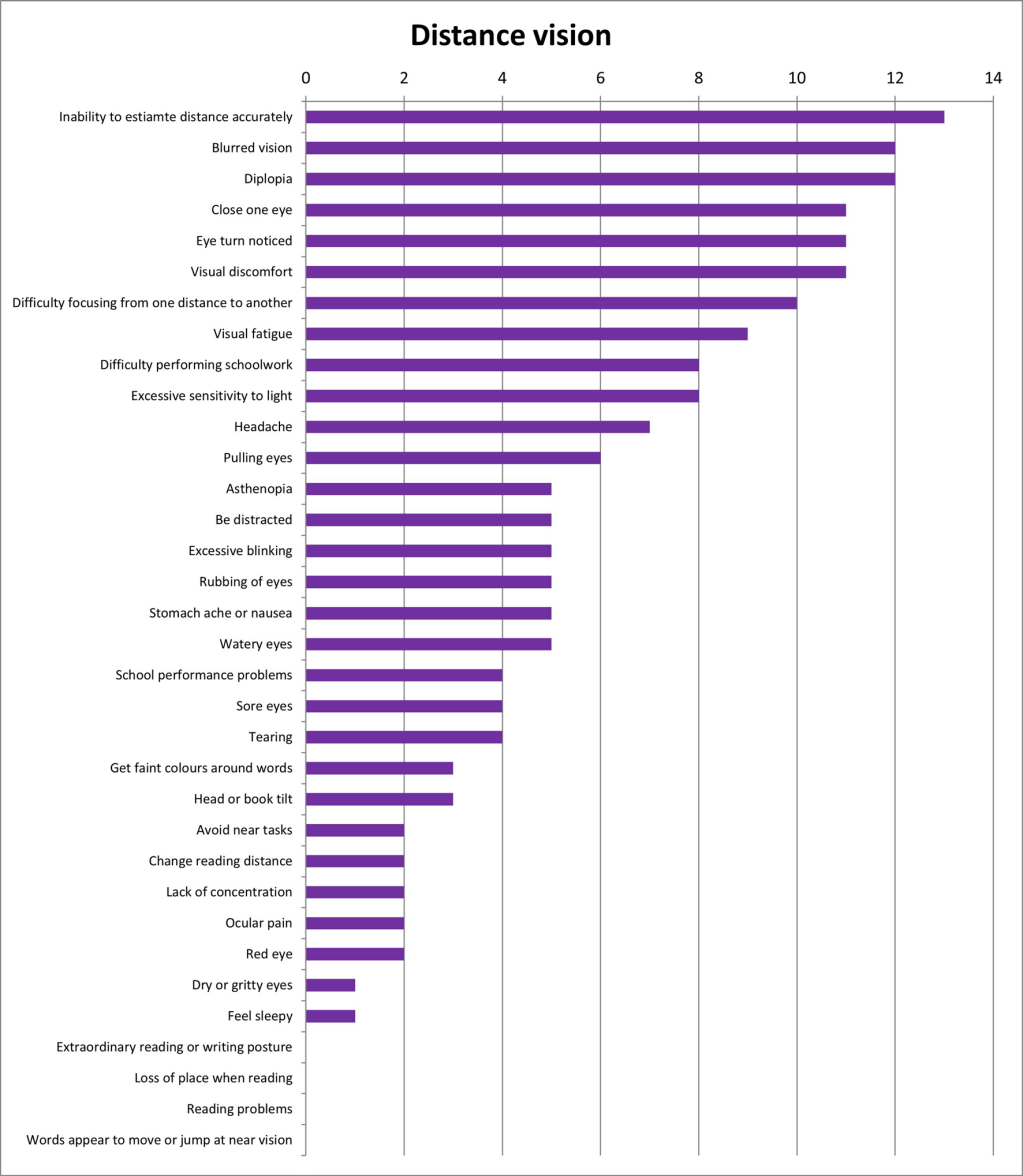
Results of round 1 for distance vision. Number of experts who associate each of 34 initial symptoms with distance vision.

**Figure 6:**
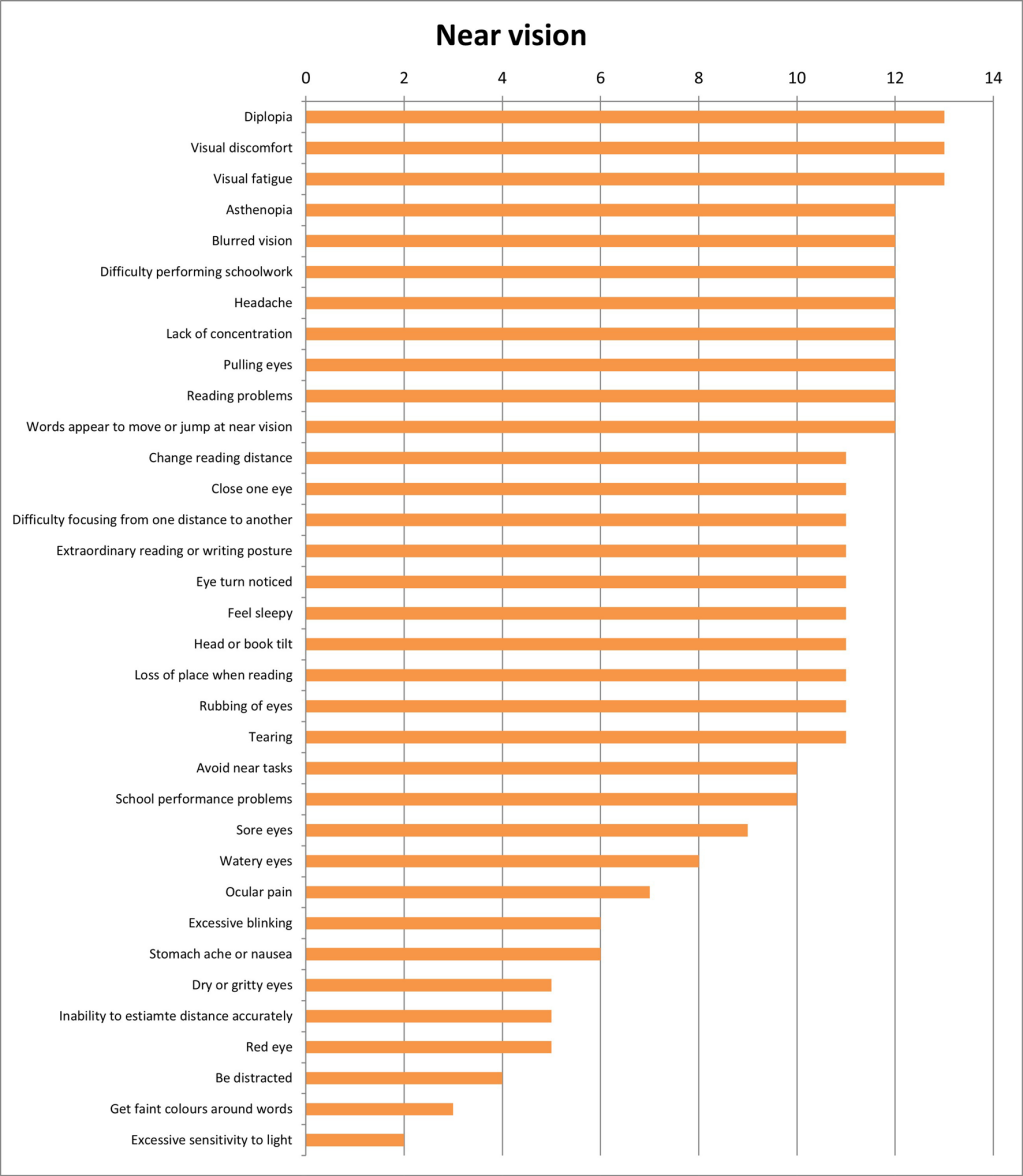
Results of round 1 for near vision. Number of experts who associate each of 34 initial symptoms with near vision.

Results of the first round were considered when the answers were designated by more or equal than 50% of experts. With these considerations, results show that 28 of the initial 34 symptoms (82.4%) were unique for refractive dysfunctions (Fig. 2); 23 of 34 symptoms (67.6%) were associated with accommodative dysfunctions (Fig. 3) and 28 of 34 (82.4%) were indicated as symptoms related to binocular dysfunctions (Fig. 4). Analysing how many symptoms overlap between categories, it is shown that 21 of 34 symptoms (61.8%) were related with the three categories of visual dysfunctions. However, only one symptom was associated with both refractive and accommodative anomalies. 5 of 34 symptoms (14.7%) were only related to both refractive and binocular dysfunctions. And there was not any symptom related to both accommodative and binocular disorders. First round results also show that experts related to distance vision 10 of 34 symptoms (Fig. 5) and most of symptoms (27 of 34 symptoms) were associated with near vision (Fig. 6).

Table 2 shows the results of the second round according to the scores given by the experts, associating each symptom with visual anomalies. Experts gave the high score to the symptom of *blurred vision* for refractive dysfunctions (9.64 ± 0.61), *difficulty focusing from one distance to another* for accommodative anomalies (9.43 ± 0.73) and *close one eye* for binocular ones (9.71 ± 0.59).

**Table 2:** Results of round 2.

	Refractive		Accommodative		Binocular	
	Mean	SD	Mean	SD	Mean	SD
Asthenopia	8.93	1.33	9.14	1.36	8.71	1.71
Avoid near tasks	7.36	2.06	7.79	1.82	7.57	2.06
Be distracted	4.93	3.13	4.57	3.92	4.86	3.68
Blurred vision	9.64	0.61	7.79	2.14	4.93	2.55
Change reading distance	8.21	1.37	8.07	2.52	6.93	2.52
Close one eye	4.29	3.30	3.71	2.68	9.71	0.59
Clumsy/ Stumble with objects	5.64	2.89	2.50	2.90	7.64	2.87
Difficulty completing assignments on time	6.93	1.71	7.64	2.02	7.64	1.95
Difficulty focusing from one distance to another	6.64	2.69	9.43	0.73	5.64	3.15
Difficulty performing schoolwork	8.14	1.41	8.21	1.70	8.21	1.61
Diplopia	4.00	2.88	3.50	3.68	9.14	1.06
Dry or gritty eyes	4.21	2.57	3.79	2.83	2.86	2.82
Excessive blinking	5.71	2.08	5.79	2.24	6.71	2.91
Excessive sensitivity to light	5.50	3.31	2.93	2.63	7.36	3.33
Extraordinary reading or writing posture	6.50	2.56	7.36	2.35	8.21	2.04
Eye turn noticed	4.00	3.40	3.50	2.99	9.57	0.73
Feel sleepy	5.50	3.35	6.14	3.48	5.43	3.22
Fixation difficulty	5.57	3.22	7.00	1.85	8.07	1.33
Forgetful	1.43	1.72	1.79	2.34	2.21	2.93
Get faint colours around words	4.29	3.30	3.21	3.41	3.07	3.24
Head or book tilt	5.00	2.27	4.00	2.45	8.36	1.76
Headache	7.92	3.22	7.79	2.04	8.21	1.78
Inability to estimate distance accurately	5.57	2.61	3.14	2.64	8.79	1.82
Inability to maintain demanding visual effort activities continuously	7.79	1.47	8.43	1.35	8.50	1.35
Irritability with visual effort activities	6.36	2.50	7.36	1.87	7.57	2.66
Lack of concentration	7.29	2.19	7.79	1.66	8.00	1.56
Loss of place when reading	5.50	2.97	6.07	2.68	8.21	1.82
Mist/Spiderweb	7.29	1.67	5.14	2.82	2.21	2.86
Neck and/or back pain	3.64	3.08	3.50	2.87	5.57	2.87
Ocular pain	5.36	2.35	5.93	2.74	4.93	2.89
Pain in the area of the eyebrows	6.00	2.20	5.57	3.06	4.86	3.00
Pulling eyes	6.64	2.19	7.07	2.52	6.00	2.88
Reading problems	6.93	2.52	7.93	2.09	8.07	2.09
Red eye	5.36	2.22	5.14	2.67	4.00	2.88
Rubbing of eyes	7.36	1.67	7.21	2.57	7.07	2.68
School performance problems	5.79	3.71	6.21	3.78	6.36	3.64
Sore eyes	5.71	2.68	5.43	2.90	4.93	3.39
Squint to see better	9.36	0.97	6.36	3.24	3.21	3.07
Stomach ache or nausea	4.29	2.37	4.57	3.20	6.86	2.87
Tearing	6.14	2.36	6.29	2.43	6.36	2.64
Visual discomfort	8.14	1.41	8.29	1.79	8.43	1.40
Visual fatigue	8.14	1.77	8.29	2.58	7.93	2.49
Watery eyes	5.93	2.43	5.64	2.72	5.71	3.49
Words appear to move or jump at near vision	6.71	2.05	7.29	2.52	6.64	3.04

Mean and standard deviation (SD) of scores given by the panel of experts, relating each of the 44 symptoms to visual dysfunctions (refractive, accommodative and binocular anomalies). Score scale from 0 to 10.

**Figure 7:**
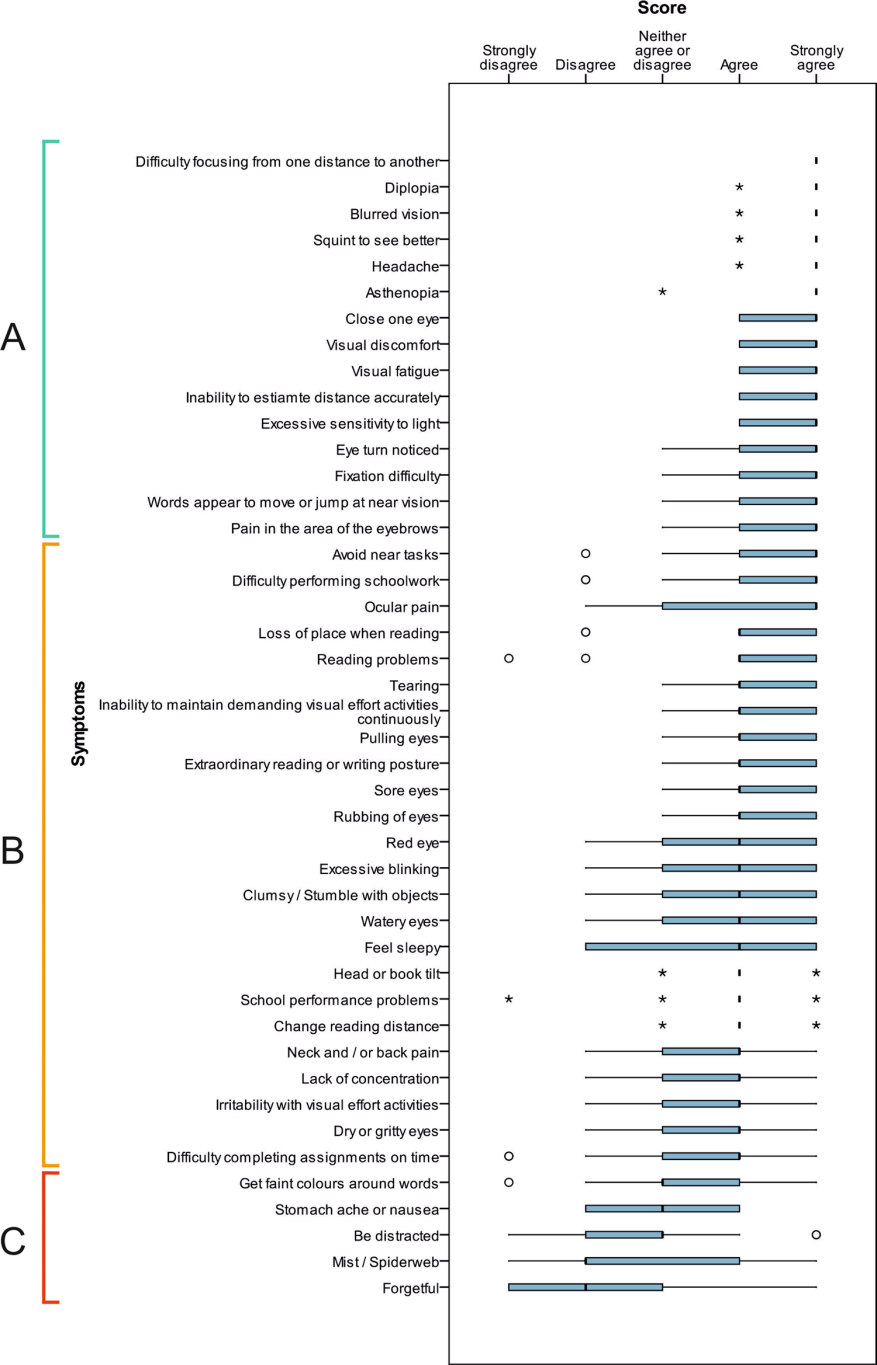
Results of round 3. Box and whisker plot showing the degree of agreement of experts regarding each of 44 symptoms to be in a visual symptom questionnaire. The width of the box shows the interquartile range (IQR). The black line in the box marks the median of the responses (which is the 50th percentile). The left line of the box shows the lower quartile (25th percentile) and the right line of the box represents the upper quartile (75th percentile). The left whisker indicates the lowest value obtained (a lower value within 1.5 IQR) and the right whisker shows the highest value obtained (which is the upper value within 1.5 IQR). The asterisk represents an outlier which is 1.5–3 IQR and the circle shows an outlier > 3 IQR. (**A**) 15 symptoms for which experts had an agreement for being in a questionnaire. (**B**) 24 symptoms with discrepancies for being in a questionnaire. (**C**) 5 symptoms for which experts had an agreement for not being in a questionnaire.

Figure 7 shows the results of the third round, in which the experts indicated their level of agreement about appearing each of the 44 symptoms in a questionnaire. Experts considered that 15 symptoms should be in a questionnaire, 5 symptoms should not appear and there was no consensus for 24 symptoms.

## Discussion

This research shows that Delphi method has been useful to identify the symptoms most likely to be associated with refractive, accommodative and binocular anomalies that should be included in a visual symptom questionnaire. The most associated symptoms with refractive anomalies were *asthenopia*, *blurred vision* and *headache*. For accommodative dysfunctions, *difficulty focusing from one distance to another* and for binocular anomalies, *diplopia* and *eye turn noticed*. Experts associated most of the symptoms to near vision. The panel of experts also gave the highest mean score to the symptom of *blurred vision* for refractive dysfunctions, *difficulty focusing from one distance to another* for accommodative anomalies and *close one eye* for binocular disorders. And they agreed with 15 symptoms should be in a questionnaire, 5 symptoms should not appear and there was no consensus for 24 symptoms.

First round results show that many symptoms related to accommodative dysfunctions are also associated with refractive anomalies, indicating that symptoms of both visual dysfunctions may be similar. This finding is similar to that observed in the study of Cacho-Martínez et al.^4^ were the authors found that uncorrected refractive error contaminates the symptoms of visual dysfunction, fundamentally when an accommodative dysfunction is present.

On the other hand, the experts associated most of the symptoms to near vision, highlighting those of *diplopia*, *visual discomfort* and *visual fatigue*. These results are similar to those obtained in the systematic review of García-Muñoz et al.^5^ in which the authors showed that most of symptoms are associated with near vision.

In the second round, when doing the analysis for dysfunctions and considering the score given by the experts, we can observe that *blurred vision* was the symptom that obtained the highest mean for refractive dysfunctions. However, it also received a high score for accommodative dysfunctions, showing that it would not be exclusive for refractive anomalies^30^, being able to be related to a refractive or accommodative anomaly^4,31–33^. This agree with the fact that the symptom of *blurred vision* appears in most of visual questionnaires^7–18^ showed by scientific literature. We can also observe for refractive dysfunctions that the symptom of *squint to see better* is the other symptom which obtained a high score and the only one in which the experts agree that is specific for refractive dysfunctions. This is one of the ten symptoms that experts added in the first round. However, the scientific evidence has shown that it only appears in two questionnaires^9,11^.

For accommodative anomalies, the highest mean was obtained by the symptom of *difficulty focusing from one distance to another* which, according to the experts, seems to be specific for these anomalies. However, it only appears in one questionnaire^16^, although this survey is related to accommodative dysfunctions. There was another symptom (*asthenopia*) which got a high mean, not only for accommodative anomalies but also, for the other dysfunctions, showing that experts gave importance to it for all visual anomalies. However, this symptom does not appear in any of existing questionnaires^7–18^.

For binocular anomalies, the symptoms with the highest mean scores were *close one eye*, *diplopia* and *eye turn noticed*. Only one of the three symptoms (that is *diplopia)* appears in 8^7–15^ out of 11 existing questionnaires^7–18^. However, the symptom of *close one eye* only appears in the 19 Item College of Optometrist in Vision Development Quality of Life (COVD-QOL)^17^ while *eye turn noticed* does not appear in any questionnaire.

In general, the symptoms *dry or gritty eyes*, *forgetful* and *get faint colours around words* had low mean scores for all anomalies. This finding may be due to the low importance the experts assigned to those symptoms.

Furthermore, results from second round show that for binocular anomalies there were more symptoms (15 that is, 34%) with mean scores greater than 8. This result is higher than that found for refractive and accommodative dysfunctions which each one had 7 symptoms (15.91%) with scores greater than 8. These results indicate that the experts gave more importance to the symptoms associated with binocular dysfunctions than refractive and accommodative ones. This situation agrees with the results found by Cacho-Martínez et al.^4^ where for clinical population it was observed a greater association between having symptoms and presenting a binocular dysfunction. In the same way, the scientific literature^5^ has also shown that most symptoms taken into account by the authors are related to binocular dysfunctions.

In any case, it is clear that the same symptom may be related to any anomaly. And although it seems that symptoms may be more associated with binocular dysfunctions, this does not imply that they are specific to a disorder. The fact that there are no specific symptoms for each visual dysfunction, suggests that a questionnaire may be developed for any visual dysfunction, and not a specific one for each visual anomaly.

In the third round, considering the established consensus, there was a great majority of agreement among experts that 15 of the initial 44 symptoms were important and should appear in a questionnaire. Figure 7 shows these symptoms. Of these 15 symptoms, 11 of them appear in most of the questionnaires published in the scientific literature^7–17^. However, the other four of them (*asthenopia*, *eye turn noticed*, *fixation difficulty* and *pain in the area of eyebrows*) do not appear in any survey. This finding shows the utility of the Delphi process, as it can be characterised symptoms commonly reported in clinical practice but not considered by the existing literature.

The experts also agreed that five symptoms were not important and should not be on the questionnaire. Of these five, two of them were included by the panel of experts (*forgetful* and *mist/spiderweb*), appearing only the symptom of *forgetful* in a questionnaire, particularly in the 19 item COVD-QOL. The remaining three symptoms (*be distracted*, *get faint colours around words* and *stomach ache or nausea*) appear in several questionnaires. The symptom of *stomach ache or nausea* is included in the CISS symptom questionnaire^13^. The symptom of *get faint colours around words* is incorporated in the Adler questionnaire^11^ and *be distracted* appears in the 19 item COVD-QOL^17^. However, the panel of experts considered that although these four symptoms are used in the scientific literature, they have little importance in the clinical setting and should therefore be omitted. This finding shows that symptoms considered by existing literature are not reported by the experts when a Delphi process is used.

For the remaining 24 symptoms, the experts had discrepancies regarding their inclusion in a questionnaire, so they should be carefully analysed for example for a future questionnaire. For this reason, those symptoms with discrepancies could be initially considered when developing a future questionnaire. Then, the appropriate psychometric methods would aid to decide to include them or not.

Of these 24 symptoms there also were discrepancies between experts who practiced in an optometric clinic and those of optical centres. Experts from optical centers, highlighted that the following 10 symptoms had a great importance when asking for dysfunctions: *clumsy/ stumble with objects, difficulty completing assignments on time*, *dry or gritty eyes*, *excessive blinking*, *feel sleepy*, *neck and/or back pain*, *ocular pain*, *red eye*, *sore eyes*, and *watery eyes*. However, experts who practiced in optometric clinics did not consider them important. This lack of agreement must be due to the different type of patients attended in each centre, but it does not have to affect to consider or not those symptoms.

When reviewing the three rounds, we can see that there is little variation in the importance that experts give to the symptoms. The 15 symptoms which experts agreed with appearing in a questionnaire, all of them were considered important in the first or second round. In addition, something similar happens with the five symptoms that the experts considered should not appear in a questionnaire. That is, symptoms as *be distracted*, *get faint colours around words* and *stomach ache or nausea* had low scores in the first and second round for all dysfunctions (although *stomach ache or nausea* obtained better score for binocular anomalies). The other two symptoms, *mist/spiderweb* and *forgetful* were not in the first round and were added by the experts in the second round. In the case of *forgetful*, the experts agreed with not appearing in a questionnaire and in fact, was not important in the first and second round. However, a particular situation occurs for the symptom of *mist/spiderweb* for which the experts considered not important to be in a questionnaire and gave low scores for accommodative and binocular disorders but an acceptable score for refractive anomalies.

The minor variation between rounds shows that from the beginning, the experts knew which symptoms could be the most important. Certainly, there were minimal variations, usual situation when carrying out a Delphi method. In fact, the importance of this method is that experts can change impressions anonymously and reflect on their position, modifying it throughout the process. In any case, the research has shown that experts had clear their position on several symptoms, with discrepancies for other symptoms.

A limitation of the Delphi method is that experts may be influenced by the scoring of others which may bias the results. To avoid this, the identities of the experts were not shown. Another limitation has been the fact that optometrists representing the entire geographical area of the country have not been included in the final panel of experts. Although the choice of experts was initially trying to cover all the possible geographical areas, there were three experts who refused to participate. In any case the geographical area which they represented were only small areas.

Despite the outlined limitations, the strength of this study is the high response rate (100%) off the experts and their good participation, as all of them answered the three rounds of the Delphi methodology.

In conclusion, this Delphi study has been useful to identify the symptoms related to visual dysfunctions, both in patients from optometric clinics and optical centers, so it can be considered the starting point to develop a tool which may facilitate the collection and classification of these symptoms. This tool could be used for diagnosing and treatment purposes.
